# Mluti-faceted interventions to prevent bloodstream MRSA infections

**DOI:** 10.1186/1753-6561-5-S6-P59

**Published:** 2011-06-29

**Authors:** PJ Orsman, JR St Andre, I Smith, L Cotter, A Gonzalez-Ruiz

**Affiliations:** 1 Infection Prevention and Control, Dartford and Gravesham NHS Trust, Dartford, UK; 2 Centre for Management of Complex Chronic Care, Edwards Hines VA Hospital, Hines. IL 60141-5151, USA

## Introduction / objectives

From 1st January until 31st December 2007, there were 31 MRSA bacteraemias at our NHS Trust. 22 were defined as HAI. It was discovered that the source of the MRSA bacteraemias in 12 (54%) of the patients was due to PIVD (Peripheral Intravascular Devices).

## Methods

The introduction of a sequence of interventions targeted at reducing MRSA bacteraemias by a newly expended infection control team, after analysis of existing polices and procedures.

The use of MRSA antimicrobials in the Trust's formulary was optimised. Teicoplanin was substituted with daptomycin in medical and surgical wards. In Intensive Care vancomycin by continuous infusion was introduced.

Introduction of MRSA screening of all adult emergency admissions and subsequent decolonisation of patients of patients found to be positive.

A new PIVD policy, insertion record and on-going care tool was launched. Skin preparation was changed from a non-sterile 70% alcohol and 0.5% chlorhexidine swab, to a sterile pre packaged application device containing 70% alcohol and 2% chlorhexidine.

Mechanical Needle-free connectors were replaced with split septum connectors.

## Results

Introduction of these 4 interventions were plotted chronologically against the cumulative MRSA bacteraemia rates and calculated per 10,000 bed days. The implementation of the 4 interventions were successful in controlling the cumulative rate of MRSA bacteraemia.

## Conclusion

The expansion of the infection control team triggered a review of procedures in our Trust. 4 Interventions were instituted to comply with best practice. Each intervention could not be shown to be effective individually, partly as the monthly rates of bacteraemias were consistently low and partly as each new intervention was introduced in quick succession.

## Disclosure of interest

None declared.

